# Effects of Artificial Intelligence Clinical Decision Support Tools on Complications Following Percutaneous Coronary Intervention

**DOI:** 10.1016/j.jscai.2024.102497

**Published:** 2025-03-18

**Authors:** Karley B. Fischer, Damian N. Valencia, Ananya Reddy, John Paul Khouzam, Ziwar F. Karabatak, Ajay Reddivari, Ammar Safar, M. Niranjan Reddy, Raja A. Nazir, Brian P. Schwartz

**Affiliations:** a Department of Internal Medicine, Kettering Health Main Campus, Kettering, Ohio; b Department of Interventional Cardiology, Kettering Health Main Campus, Kettering, Ohio; c Boonshoft School of Medicine, Wright State University, Dayton, Ohio

**Keywords:** acute kidney injury, artificial intelligence, contrast nephropathy, percutaneous coronary intervention

## Abstract

**Background:**

Artificial intelligence (AI) models have been created that incorporate unique patient characteristics to risk stratify patients undergoing cardiac catheterization with percutaneous coronary intervention (PCI). The most frequent complications following PCI are contrast-induced acute kidney injury (CI-AKI) and postprocedural bleeding, resulting in increased adverse outcomes, length of stay (LOS), and health care costs. Our study investigates the impact of AI clinical decision support tools on these events.

**Methods:**

A retrospective review of patients undergoing PCI at our institution from April 2023 to March 2024 was performed. All patients had an ePRISM (Terumo Health Outcomes AI clinical decision support tool) generated risk assessment and maximum contrast volume recommendation reported during procedure time-out. Statistical analysis was performed to determine the incidence of post-PCI CI-AKI, bleeding events, and LOS.

**Results:**

A total of 642 patients were analyzed. The incidence of CI-AKI significantly declined from a baseline of 10% to an average of 2.18% (*P* < .0001). Of the remaining CI-AKI, 92.9% occurred in hospitalized patients. The incidence of bleeding complications declined from a baseline incidence of 2.15 per month to an average of 1.54 per month. Our institution’s average LOS declined from a baseline of 3.44 to 1.79 days.

**Conclusions:**

AI clinical decision support tools can be effectively incorporated into clinical practice. ePRISM successfully risk-stratified patients undergoing PCI for CI-AKI and bleeding events and gave meaningful recommendations which resulted in a significant reduction in adverse events and LOS.

## Introduction

Contrast-induced acute kidney injury (CI-AKI) is the leading cause of iatrogenic acute nephropathy, occurring in 7% of patients undergoing coronary angiography with percutaneous coronary intervention (PCI).^1^ Acute kidney injury (AKI), defined by the Kidney Disease International Global Outcomes Guidelines, is a rise in serum creatinine of ≥0.3 mg/dL within 48 hours of contrast media exposure or ≥50% elevation from baseline.^1^ The incidence is greater in patients with chronic kidney disease (CKD), occurring in up to 30% of patients post PCI^2^^,^^3^ and in patients presenting with ST-elevation myocardial infarction (STEMI) or developing cardiogenic shock where the risk more than doubles. CI-AKI results in increased adverse outcomes, length of stay (LOS), and health care costs. Severe CI-AKI also results in a graded increase in the risk of bleeding complications, the need for renal replacement therapy or transplant, and death.^1^

CI-AKI does not have definitive treatment; thus, prevention is paramount. Traditionally, prevention methods included periprocedural hydration and avoidance of renal toxic pharmacologic agents in the periprocedural period.^4^ Prevention strategies continue to broaden with advancements in technology. Alternative imaging modalities including intravascular ultrasound and optical coherence tomography reduce the volume of contrast required by allowing coronary artery lesion visualization via alternative methods.^5^ Radial artery access, due to decreased bleeding risk and subsequent hemodynamic instability, has been associated with reduced CI-AKI.^6^ The optimal volume of contrast media that can be safely used has been an ongoing debate among operators in the cardiac catheterization laboratory. The Mehran risk prediction score is 1 validated method, based on factors including age, hemoglobin, preexisting CKD, need for intraaortic balloon counterpulsation, and numerous additional variables.^1^ However, this risk prediction calculator only includes postprocedure variables that are solely known following case completion in the catheterization laboratory. Although it is a valuable retrospective analysis tool for better understanding which patients develop CI-AKI, it cannot prospectively identify high-risk patients because it does not include variables that can be anticipated before the procedure. The NCDR Reduced AKI model successfully predicted risk with preprocedural variables only but does not provide the personalized safe volume of contrast recommendations or directly interface with the electronic health record for seamless integration into the procedural workflow.^7^ More contemporary algorithms have been studied; however, the risk categorization was rather broad and defined patients as those with high risk with recommended contrast <20 mL, those with modifiable risk with recommended contrast 20 to 500 mL, and those with low risk with recommended volume of contrast >500 mL.^8^ The majority of the patient population will fall in the modifiable risk while undergoing a procedure that in the majority of cases uses contrast in the range of 20 to 500 mL. Therefore, the model fails to further categorize a large number of patients who could benefit from more patient-specific and targeted recommendations for safe volume of contrast. Models have retrospectively been created to begin analyzing patient-specific volumes of contrast that can safely be used, but have not been implemented into software that interfaces with the electronic health record to allow incidence to be analyzed while following recommendations at the time of procedure.^9^

Bleeding complications post-PCI have historically been difficult to fully understand and change, as there are more than 10 definitions for major bleeding events used in various trials and registries.^10^ Prognosis, however, has consistently been directly related to bleeding events, with increased 30-day mortality across all trials.^11^ The most frequent procedure-related complication historically has been access site bleeding.^11^ With increasing operator preference for radial artery access rather than femoral artery access, access site bleeds have decreased in recent decades in patients undergoing PCI.^12^ However, in cases with complex PCI with the use of device therapy or simultaneous placement of structural devices, a return to femoral artery access becomes necessary. Retroperitoneal bleeding following femoral artery access carries higher mortality, similar to that of nonaccess site bleeding.^10^

Gastrointestinal bleeding (GIB) is the most frequent noncardiac complication following PCI, with an in-hospital mortality of up to 10%.^11^ Independent predictors of GIB include age, tobacco use, triple antithrombotic therapy, and history of malignancy.^11^ The incidence of GIB is often underestimated, as the recommended use of dual antiplatelet therapy following PCI with drug-eluting stent for 1 year is the greatest risk factor, whereas studies typically focus on in-hospital or 30-day post-PCI events.^11^

Length of stay following coronary angiography with PCI has fortunately trended downward with improvements in health care technology, procedural techniques, and pharmacotherapy.^13^ LOS directly contributes to treatment costs, affecting both the patient and health care system at large. Further decreasing LOS became an important factor during the COVID-19 pandemic, when shortages of hospital bed availability and staff became a national challenge. Early discharge when appropriate became widely acceptable. However, a further decrease in average LOS is dependent upon an increase in the number of patients with no or minimal complications, thus stable for early discharge without compromising patient safety. Patients with CI-AKI or bleeding complications are typically not ready for discharge in the same time frame as patients without complications; thus, a reduction in complication rate should correlate with a reduction in average LOS.

Artificial intelligence (AI) models have been created which incorporate unique patient characteristics, in order to provide patient-specific CI-AKI risk and contrast recommendations. ePRISM (Terumo Health Outcomes AI clinical decision support tool) is a prospective AI algorithm that assesses a patient’s unique risk for AKI based on 23 different variables. ePRISM technology was developed by Terumo Health Outcomes as an AI clinical decision support tool. The software model is a weighted multiple-variable model based on proprietary risk modeling by Terumo. Patients are classified as those with low risk, moderate risk, or high risk. Based on AI analysis of unique risk factors and risk class, a maximum recommended volume of contrast is calculated. During preprocedural time-out, the maximum volume of contrast is reported. The AI algorithm applies multipliers to many of the known contributing variables such as age, sex, left ventricular dysfunction, and prior diabetes mellitus or kidney disease. The utility for prevention with such AI tools is that all of the utilized data are known preprocedure, allowing the risk to be known and acted on prior to and during coronary angiography. We sought to determine the impact AI can have on CI-AKI by analyzing the incidence of CI-AKI, bleeding complications, and LOS after initiating the ePRISM clinical decision support tool in our cardiac catheterization laboratories.

## Methods

This is a single center (Kettering Health Main Campus in Kettering, Ohio) institutional review board–approved retrospective observational analysis on patients who underwent coronary angiography with successful PCI between April 1, 2023, and March 31, 2024. All subjects undergoing coronary angiography with the use of ePRISM in the designated time period were screened. Patients who had undergone successful PCI were selected and outcomes were assessed to determine if the ePRISM generated risk assessment and maximum contrast volume recommendation reported during procedure time-out could reduce the incidence of CI-AKI, bleeding complications, and LOS from baseline. All patients in this series underwent coronary angiography with PCI for appropriate use criteria indications and provided informed consent prior to the procedure.

ePRISM was integrated with the electronic health record (Epic Systems), allowing for automatic data input from the patient’s historical and preprocedural workup. Physician workflow had minimal impact. Preoperatively, nursing staff reviewed the patient data, noted the calculated ePRISM risk assessment, and were the point of contact with the patient to provide education regarding medications to hold perioperatively, necessary hydration, and if indicated early arrival for prehydration. On the day of the procedure, the operating physician was informed of the ePRISM risk assessment and notified regarding preprocedure hydration if given. ePRISM-generated risk assessment and recommendations regarding CI-AKI and bleeding complications were stated during preprocedural time-out, with the recommended contrast limit. End diastolic pressure was obtained to determine if additional IV fluid bolus or maintenance fluids were indicated. Intraoperatively, a reminder was provided to the physician when approaching 80% of the recommended ePRISM contrast limit. Based on the total contrast used, postprocedure fluids were decided.

ePRISM provided each patient with an AI personalized risk assessment for developing post-PCI CI-AKI, categorized as low, moderate, or high risk. Low risk correlated with <4% risk of post-PCI CI-AKI. Moderate risk correlated with 4% to 7% risk of post-PCI CI-AKI. High risk correlated to >7% risk of post-PCI CI-AKI. Based on risk, AI provided a maximum contrast volume recommendation. Incidence of CI-AKI among patients was recorded along with factors including calculated risk for CI-AKI and risk class (low, moderate, high), inpatient versus outpatient procedure, volume of contrast recommended, and volume of contrast used by the operator.

ePRISM provided each patient with an AI personalized risk assessment for developing post-PCI bleeding complications, categorized as low, moderate, or high risk. Low risk correlated with <2% risk of post-PCI bleeding events. Moderate risk correlated with 2% to 5% risk of post-PCI bleeding events. High risk correlated with >5% risk of post-PCI bleeding events. Incidence of bleeding events among patients was recorded along with factors including calculated risk for bleeding events and risk class (low, moderate, high). Bleeding events were categorized as access site bleeding, GIB, cardiac tamponade, and intracranial bleeding. Focus on risk was emphasized to physicians beginning in August 2023.

Data regarding postprocedure LOS were collected. Patient procedural and demographic data were deidentified and provided to the investigators, who performed statistical analysis at Kettering Health Network.

Statistical analysis was performed to determine the incidence of post-PCI CI-AKI, post-PCI bleeding events, as well as LOS and to track variance with the implementation. The results were compared to control group baseline data averaged over the prior 1 year to implementation of the use of the ePRISM analysis and recommendations. Incidence preinitiation and postinitiation were analyzed with an incidence rate ratio. Categorical variables are expressed as frequencies and percentages.

## Results

A total of 642 patients who underwent coronary angiography with successful PCI, including both inpatient and outpatient, with the use of ePRISM prior to procedure start time were analyzed. The average age was 68.1 years at the time of the procedure, 68.8% were male, and 39.1% were hospitalized patients.

In regard to risk for CI-AKI, 37.2% of patients were classified as those with low risk (<4%), 36.5% with moderate risk (4%-7%), and 26.3% with high risk (>7%) for post-PCI CI-AKI (Table 1).

**Table 1:** ePRISM-generated CI-AKI and bleeding event risk assessment in patients prior to coronary angiography with percutaneous coronary intervention

	Low	Moderate	High
CI-AKI risk assessment	37.2%	36.5%	26.3%
Bleeding event risk assessment	25.2%	63.7%	11.1%

CI-AKI, contrast-induced acute kidney injury.

**Figure 1:**
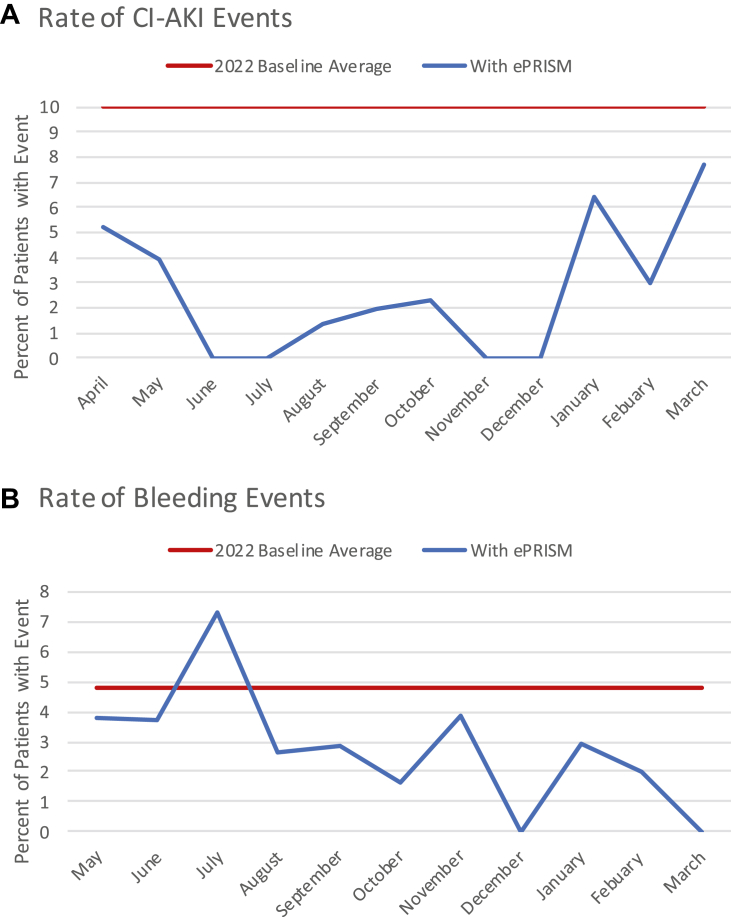
**Trends of events with ePRISM**. (**A**) Incidence of contrast-induced acute kidney injury (CI-AKI). (**B**) Incidence of bleeding events.

Following initiation of ePRISM in our institution, the incidence of CI-AKI significantly declined from a baseline of 10% per month to an average of 2.18% per month in the first 12 months of protocol initiation (Figure 1A). The incidence rate ratio was 4.59 (*P* < .0001). There was a nearly 80% reduction in PCI-associated CI-AKI with the implementation of ePRISM recommendations.

There were 14 remaining CI-AKI events. The average age of patients who developed CI-AKI was 71.7 years, and 85.7% were male. The average risk of the 14 patients was 10.1%, characterized as those with high risk. Of the remaining CI-AKI, 92.9% occurred in hospitalized patients. The ePRISM recommended contrast maximum was exceeded in 71.4% of the remaining cases of CI-AKI. The average volume of contrast used in patients who developed CI-AKI was 116.6 mL, ranging from 70 to 190 mL. No patients required dialysis following CI-AKI.

A total of N = 584 patients were analyzed for bleed risk from May 2023 to March 2024; 26.2% were classified as those with low risk, 62.6% as those with moderate risk, and 11.2% as those with high risk for bleeding complications (Table 1). The incidence of bleeding complications was clinically significant with a decline from a baseline of 2.15 to 1.54 (Figure 1B), reducing the average from 4.8% to 2.9%. The incidence rate ratio was 1.64 (*P* = .1038). There was a 40% relative risk reduction in postprocedure bleeding events.

**Figure 2:**
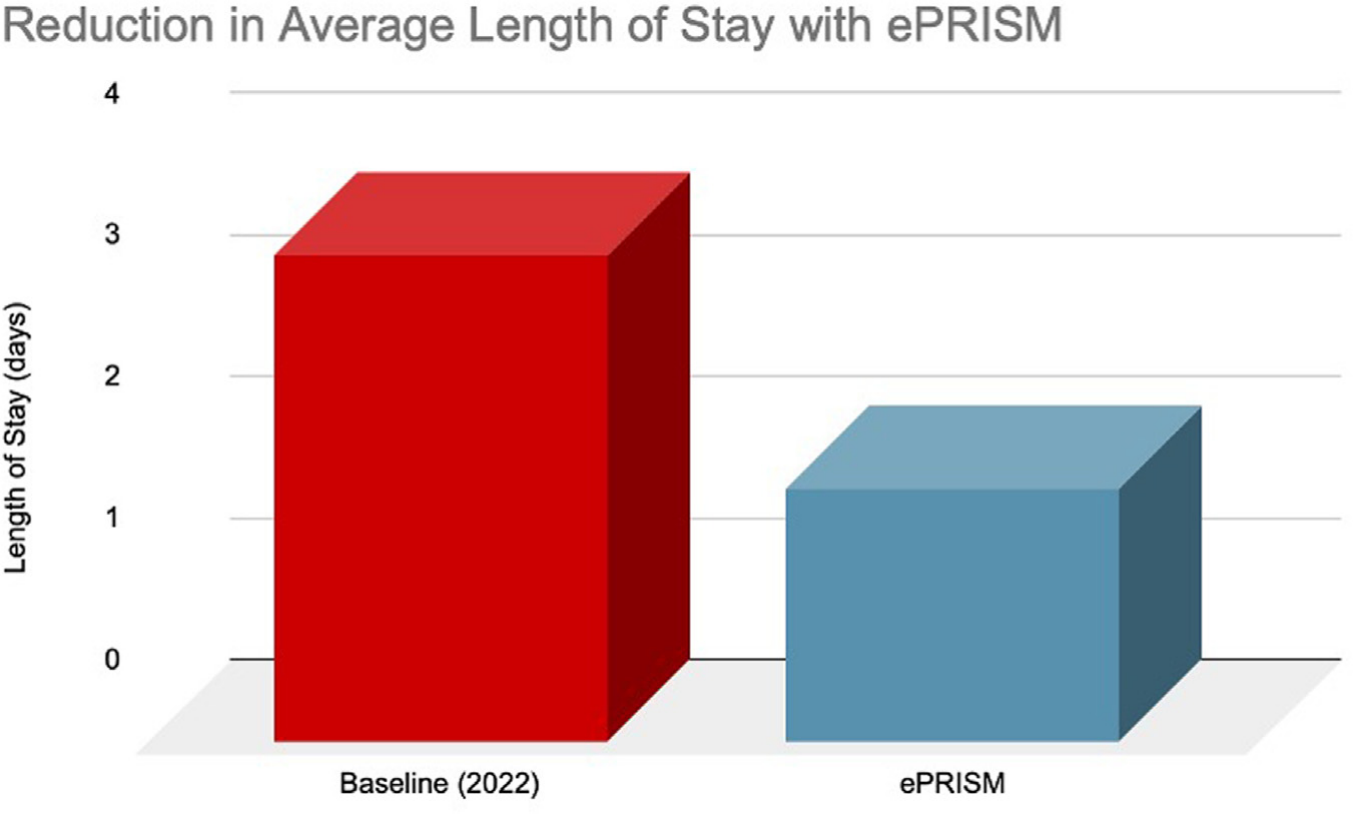
**Impact of ePRISM on length of stay.**

Following the initiation of ePRISM in our institution, average LOS significantly declined by 48%, from a baseline of 3.44 days to 1.79 days in the first 12 months of protocol initiation (Figure 2). Patients with reduced average LOS of 1.79 days were subcategorized as those with CI-AKI and without CI-AKI, where LOS was 6.3 days and 1.68 days, respectively.

## Discussion

In this study, we found that the use of AI via ePRISM resulted in a significant decrease in the incidence of CI-AKI and bleeding complications in patients undergoing PCI. The average LOS subsequently decreased (Central Illustration). AI is likely to continuously improve with larger data sets and patient information to further improve patient outcomes. Although a history of diabetes mellitus and CKD are known to be the primary risk enhancers, AI has the ability to apply multipliers and identify patients who may not have the primary risk factors that are easily recognized as moderate to high risk.^2^^,^^3^ Frequently, patients have multiple conditions or factors that each slightly increase risk, which can be easily overlooked in procedure preparation. However, AI can quantify these risks and recognize the synergistic effects that contribute to the risk of CI-AKI. This quantification of risk brought to attention during the periprocedural timeframe, results in active decision-making by the operator including access site and volume of contrast decisions.

**Central Illustration:**
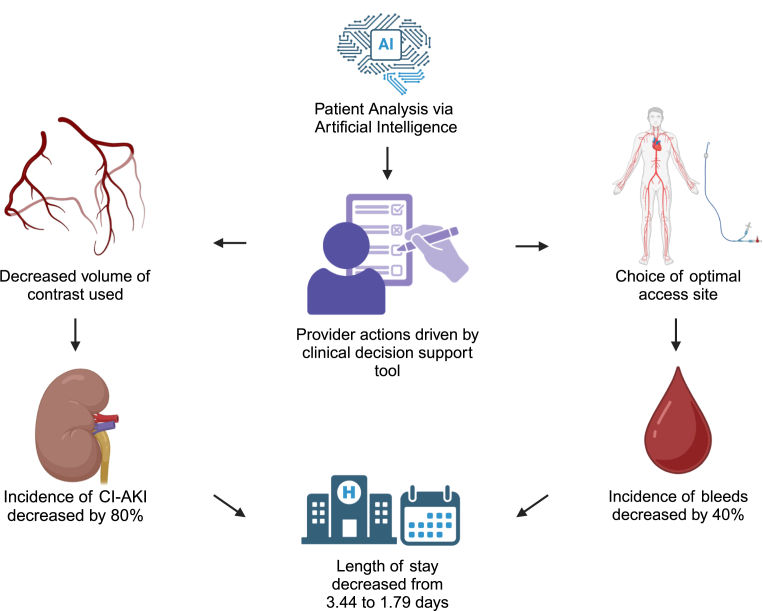
**The impact of ePRISM artificial intelligence clinical decision support tool on patients undergoing cardiac catheterization with percutaneous coronary intervention.** CI-AKI, contrast-induced acute kidney injury.

Following implementation, a large majority of the remaining incidence of CI-AKI in our study occurred in hospitalized patients, which is speculated to be correlated to critical illness at the time of PCI, additional contrast exposure precardiac and postcardiac catheterization laboratory for noncardiac imaging, increased use of renal toxic medications, and derangements in hydration status associated with hospitalization. Further advancement of AI may allow for the integration of factors such as recent contrast exposure into the algorithm or larger modifiers correlating with inpatient status. The ePRISM-recommended amount of contrast was exceeded in a large majority of these cases. A higher proportion of patients in this group presented with STEMI or cardiogenic shock. Therefore, it may have been necessary to continue the procedure with larger volumes of contrast, rather than planning for additional outpatient staged PCI. Second, both STEMI and cardiogenic shock are associated with higher rates of post-PCI CI-AKI.^1^

The contrast was more often to be exceeded in high-risk patients, where the recommended volume of contrast was typically less than 50 mL. Following risk-benefit discussions, the necessity to effectively address the significant lesion required exceeding the recommended volume of contrast. Although technology has improved the ability to complete a selective coronary angiogram with PCI using lower volumes of contrast than historically required, the ability to comply with strict low volumes remains a challenge. As intravascular ultrasound, optical coherence tomography, or similar alternatives become more widely used, the necessary volume of contrast could further decrease. In patients predicted to be at high risk with the volume of contrast recommended inadequate to complete the procedure, it may be beneficial to incorporate the use of high-resolution dynamic imaging and intravascular imaging to further reduce CI-AKI.

The incorporation of volume contrast into the procedure time-out brings awareness to the operator that can consciously and subconsciously contribute to the judicious use of each bolus of contrast injected.

Due to the multifactorial causes of PCI-related CI-AKI including events such as hypotension, fluid shifts, and plaque embolization, complete prevention of AKI post-PCI is unlikely.^6^ However, reduction of CI-AKI by reducing the volume of contrast media used to a personalized level is considered to be safer based on patient characteristics and can significantly reduce morbidity and mortality associated with post-PCI adverse events secondary to contrast media. A randomized clinical trial proved a similar concept of clinical decision support tools regarding contrast volume and hemodynamic-guided intravenous fluid targets, with an absolute risk reduction of 2.3%.^14^ Our study further validates the use of the ePRISM tool outside of a clinical trial, in a community hospital with a clinically and statistically significant reduction in incidence of CI-AKI, and a decrease in LOS and complications associated with the procedure.

The AI bleeding risk protocol also resulted in meaningful access site decisions based on risk stratifications. Initiation of the protocol resulted in a significant reduction in adverse bleeding events. The choice of access site was an active decision made at the time of the procedure. The majority of operators in our institution preferentially gain radial access when feasible and not contraindicated. In addition, the risk of bleeding events was at times compared to the risk of CI-AKI when making decisions on proceeding with the treatment of additional lesions thus exceeding the recommended contrast volume, versus staging PCI due to a patient categorized with high bleed risk for undergoing arterial puncture.

A significant decrease in LOS is likely the result of reduced periprocedural complications. A decrease in LOS results in improved cost-effectiveness and a reduction in-hospital resources.

### Study limitations

Due to the retrospective nature of this analysis and the use of data from a single center, this study may not be representative of the general population. A larger multicenter analysis using a similar algorithm is necessary to increase data strength. This study only investigated patients who underwent PCI, and this may have confounding factors such as new initiation of antiplatelet therapy. High-risk PCI and PCI in the setting of acute coronary syndrome increase the incidence of AKI and bleeding events; therefore, the inclusion of these populations may underestimate the reduction of events. Therefore, data results may not represent all patients undergoing cardiac catheterization.

### Clinical perspectives

Post-PCI incidence of CI-AKI and bleeding events results in adverse patient events and increased LOS. AI clinical decision support tools can be successfully used in patients undergoing PCI to effectively and efficiently guide the volume of contrast that can be safely used as well as access site recommendations. The use of AI can result in a significant statistical and clinical reduction in adverse events that ultimately reduce LOS and overall health care costs. Patient care is optimized by reducing adverse outcomes and safely discharging patients home faster. The use of AI in health care will continue to increase, thus efficiently providing physicians recommendations by analyzing large volumes of data rapidly. Although previously developed models have categorized patient risk, the recommended volume of safe contrast provided was a broad range rather than a patient-specific target that ePRISM provided, which allowed physicians to have an exact number to avoid exceeding. Knowing that a patient is at risk at 95 mL, is more achievable than knowing that a patient is at risk anywhere from 20 to 500 mL.^8^ Increased patient data in the AI models will continue to improve sensitivity and accuracy to effectively predict events that can be prevented by appropriate physician decision-making. A further understanding of why events occurred that were not predicted by the model, is necessary to continue improving the utility of AI. Ultimately, preprocedural prospective use of AI models in a registry is warranted to obtain multicenter outcomes to determine if results are similar to those studied in a prior clinical trial and our institution.^14^

## Conclusion

AI clinical decision support tools can effectively be incorporated into clinical practice to augment provider expertise and decision-making. ePRISM was successfully able to risk stratify patients undergoing PCI for CI-AKI and provide meaningful recommendations in terms of maximum contrast volume which resulted in a drastic reduction in patient adverse events including CI-AKI and bleeding events. The average LOS was also positively impacted.
